# Highly regional generation and heterogeneous differentiation of basal cells in mouse trachea

**DOI:** 10.1038/s41421-026-00887-4

**Published:** 2026-03-30

**Authors:** Xiuxiu Liu, Wendong Weng, Zhen He, Kuo Liu, Maoying Han, Wenjuan Pu, Bin Zhou

**Affiliations:** 1 CAS CEMCS-CUHK Joint Laboratory, New Cornerstone Science Laboratory, State Key Laboratory of Cell Biology, Shanghai Institute of Biochemistry and Cell Biology, Center for Excellence in Molecular Cell Science, Chinese Academy of Sciences, University of Chinese Academy of Sciences, Shanghai, China; 2 Key Laboratory of Systems Health Science of Zhejiang Province, School of Life Science, Hangzhou Institute for Advanced Study, University of Chinese Academy of Sciences, Hangzhou, Zhejiang, China; 3 School of Life Science and Technology, ShanghaiTech University, Shanghai, China

**Keywords:** Cell division, Cell growth

Dear Editor,

The trachea connects the lungs to the external environment as part of the respiratory system. Its ventral side is supported by a C-shaped cartilage, while the dorsal side contains smooth muscle. Inside, the pseudostratified epithelium consists mainly of basal cells, secretory cells, and ciliated cells^1^. Basal cells function as the primary stem cell population, maintaining tissue balance through self-renewal and differentiation^2^. Although epithelial turnover is typically low under normal conditions^3^, basal cells exhibit remarkable proliferative responses to injury^2,4^. Understanding how basal cells are regulated is, therefore, key for respiratory health and therapy development.

Extensive research has revealed functional subgroups among basal cells. For instance, those with high *Krt5* promoter activity show greater in vitro growth^5^, and *Krt14-CreER*-labeled basal cells displayed high self-renewal potential^6^. Combining *Krt5-CreER* tracing with modeling identified two distinct types: multipotent basal stem cells with symmetric division potential and basal luminal progenitors differentiating into secretory lineages^3^. Single-cell RNA sequencing (scRNA-seq) also identified *Krt13*-expressing cells in the trachea hillock with high proliferation potential^7^. This heterogeneity is established during early development^8^ and persists in adulthood. Notably, dorsal basal cells show greater in vitro colony formation ability, while ventral counterparts differentiate more readily after injury^9,10^.

While these findings support basal cell heterogeneity, regional heterogeneity lacks definitive in vivo genetic validation. Current lineage tracing approaches using specific markers may introduce selection bias. Importantly, although basal cell proliferation varies by context, the fundamental proliferative dynamics and turnover rates of the basal cell population under physiological conditions remain poorly characterized.

To assess tracheal epithelial cell proliferation patterns in vivo, we firstly employed the ProTracer system^11^, generating *R26-DreER;Ki67-CrexER;R26-GFP* triple transgenic mice (Supplementary Fig. S1a). Tamoxifen (Tam) administration induces DreER nuclear translocation, which excises the *rox*-flanked estrogen receptor (ER) element from the *Ki67-CrexER* cassette and converts it into functional *Ki67-Cre*, enabling permanent GFP labeling of proliferating cells through Cre-loxP recombination^12^. Wholemount imaging of tracheae revealed minimal GFP signal at day 3 post-Tam, but progressively intensified from day 7 to week 4, with pronounced dorsal enrichment (dotted lines, Supplementary Fig. S1b, c). No leakiness was observed in controls (Supplementary Fig. S1d). Cross-section analysis confirmed GFP^+^KRT5^+^ basal cells in both dorsal and ventral regions, though dorsal proliferation significantly exceeded ventral, with a similar disparity in KRT8^+^ luminal cells (Supplementary Fig. S1e, f). At 4 weeks post-Tam, secretory (CC10^+^) and ciliated (FOXJ1^+^, A-Tub^+^) lineages also showed regional GFP labeling bias (Supplementary Fig. S1g–k). EdU pulse labeling independently validated dorsal-ventral basal cell proliferation asymmetry (Supplementary Fig. S1l, m). While ProTracer labeled most proliferating cell types, immune cells (CD45^+^) were detected, whereas endothelial cells and fibroblasts were rare (Supplementary Fig. S1n). Collectively, these data demonstrate that tracheal epithelial proliferation, particularly in basal cells, occurs at significantly higher rates dorsally than ventrally during homeostasis.

Given the critical role of basal cells as a stem cell reservoir in homeostasis and repair, we focused on their proliferation dynamics. To specifically monitor basal cell turnover, we developed a functional ProTracer (fProTracer)^13^ system by employing *P63-CreER* to prime basal cell-specific proliferation (Fig. 1a). The basal cell fProTracer (BC-fProTracer) system was generated with *P63-CreER*;*Ki67-loxP-Stop-loxP-Dre* (*Ki67-L-Dre*)*;R26-rox-Stop-rox-loxP-Stop-loxP-GFP* (*R26-RL-GFP*) triple genetic mice. Upon Tam treatment, CreER in basal cells translocated to the nuclei, mediating Cre-*loxP* recombination both in *Ki67-L-Dre* and *R26-RL-GFP* alleles, converting them into *Ki67-Dre* and *R26-R-GFP* respectively. Subsequent basal cell proliferation was then recorded by Ki67-derived Dre-rox recombination. Validation confirmed efficient basal cell targeting without leakiness and cross-talk (Supplementary Fig. S2a, b). In BC-fProTracer mice, whole-mount imaging revealed time-dependent GFP accumulation from days 3 to 28 post-Tam, predominantly in dorsal regions (Fig. 1b and Supplementary Fig. S2c). Immunostaining at 4 weeks confirmed GFP labeling in KRT5^+^ basal cells but not in KRT8^+^ luminal cells, with dorsal predominance (Fig. 1c, d). GFP-labeled secretory cells and rare ciliated cells were also observed, revealing that basal cell division generates secretory cells within one month (Supplementary Fig. S2d–f). Discrepancies between ubiquitous ProTracer and BC-fProTracer reveal distinct contributions: basal cell proliferation (captured by both) and luminal cell self-proliferation (captured only by BC-fProTracer) (Supplementary Fig. S1g–i, d–f). The serial visualization of the captured GFP signals revealed a basal cell proliferating to generate a secretory cell (Supplementary Fig. S2g). This provides evidence that secretory cells could be generated by basal cells through asymmetrical division. GFP^+^NOTCH3^+^ (a reported basal luminal progenitor marker^14^) cells were also observed, indicating basal cells give rise to basal luminal progenitors in both regions (Supplementary Fig. S2h).

**Fig. 1:**
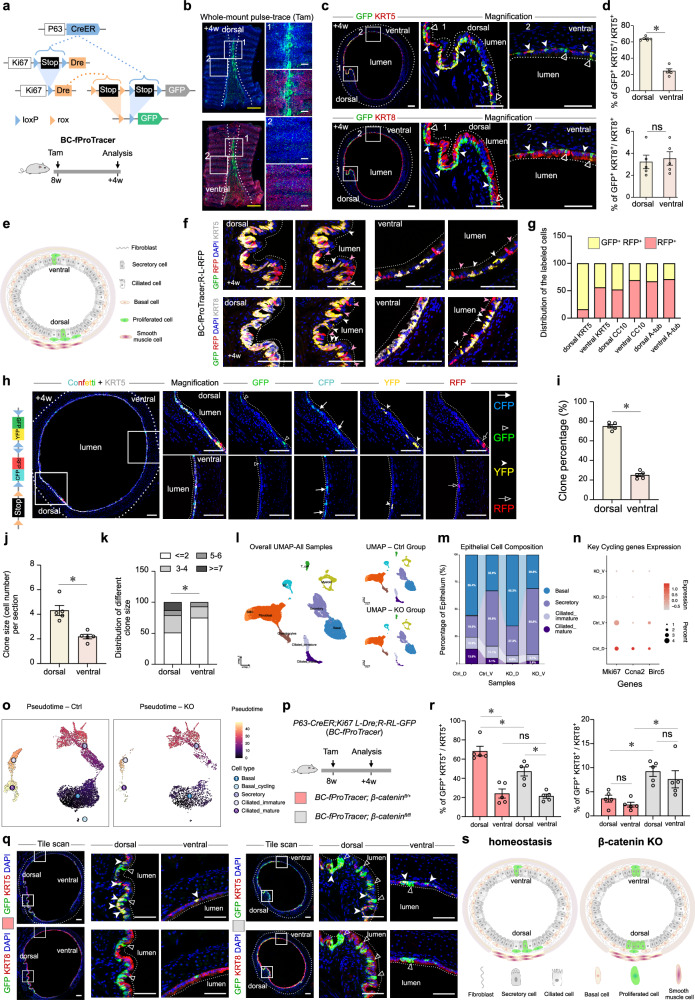
Lineage tracing reveals spatial heterogeneity in trachea basal cell proliferation and identifies two WNT-regulated subpopulations responsible for proliferation and differentiation. **a** Schematic of the BC-fProTracer strategy (*P63-CreER;Ki67-L-Dre;R26-RL-GFP*) for basal cell-specific proliferation tracing. **b** Whole-mount staining (A-Tub, GFP) in BC-fProTracer mice at 4 weeks post-Tam. **c** Cross-trachea sections 4 weeks post-Tam stained for GFP with basal cell marker KRT5 or luminal marker KRT8. Arrowheads indicated KRT5^+^GFP^+^ basal cells; Hollow arrowheads indicated KRT8^+^GFP^+^ luminal cells. **d** Quantification of GFP^+^ basal and luminal cells in dorsal versus ventral regions. **e** Model cartoon images: basal cell proliferation generates new basal (self-renewal) and luminal cells. Dorsal enrichment of proliferated basal cells is shown. **f** Cross-trachea sections collected 4 weeks post-Tam from *P63-CreER;Ki67-L-Dre;R-RL-GFP/L-RFP* mice were stained with KRT5 and KRT8. White arrowheads indicate GFP^+^RFP^+^ cells, while red arrowheads indicate RFP^+^ cells. **g** Quantification of the proportion of GFP^+^RFP^+^ cells versus RFP^+^ cells in multiple lineages. **h** Confetti^+^ basal cell clones (KRT5^+^) in coronal sections 4 weeks post-Tam. Split channels show distinct clones. **i** Quantification of clone distribution (dorsal vs ventral). **j** Quantification of average clone size. **k** Clone size distribution in dorsal vs ventral regions (*χ*^2^ test). **l** scRNA-seq identification of cell populations. UMAP visualization illustrates cell-type composition across ctrl and KO tracheal samples. **m**, **n** Changes in epithelial subtypes. **m** Stacked bar chart of cell composition. **n** Key cycling genes in the Ctrl and KO groups. **o** UMAP visualization of pseudotime analysis. **p** Schematic combining BC-fProTracer with conditional *β-catenin* knockout. **q** Trachea sections stained for KRT5 (basal), KRT8 (luminal), and GFP. Arrowheads: GFP^+^KRT5^+^ basal cells; Hollow arrowheads: GFP^+^KRT8^+^KRT5^–^ luminal cells. **r** Quantification of GFP^+^ basal and luminal cells in control and knockout (cKO) mice. **s** Cartoon models: β-catenin promotes basal cell self-renewal (symmetrical division) and inhibits differentiation or division into luminal cells. Statistical analysis: Unpaired *t*-test; * *P* ≤ 0.05; ns, *P* > 0.05. Data represent mean ± SEM. Scale bars: yellow, 1000 µm; white, 100 µm.

Given the reported high proliferation rate of KRT13^+^ hillock cells^7^, we assessed their proliferation labeling. Immunostaining for GFP, KRT13, and KRT5 identified KRT13^+^ hillock cells and another KRT13^–^KRT5^+^ non-hillock basal cells. Both showed comparable GFP labeling at 4 weeks (Supplementary Fig. S3a, b), indicating that high proliferative capacity is not restricted to KRT13^+^ hillock cells. Short-interval sampling after Tam revealed proliferation of KRT13^–^KRT5^+^ basal cells both around and distal to hillocks at day 2 (Supplementary Fig. S3c). Quantification of the 7- and 14-day samples revealed that, although their proliferation rate was slightly lower than that of hillock cells, sustained proliferation of KRT13^–^ basal cells nevertheless contributed to the dorsal-ventral regional heterogeneity (Supplementary Fig. S3d, e).

Collectively, BC-fProTracer tracing demonstrated that dorsal basal cells exhibited significantly higher self-renewal activity than ventral counterparts during homeostasis, with no regional difference in luminal generation within this one-month timeframe (Fig. 1e). By integrating the R26-L-RFP reporter with BC-fProTracer, we directly tracebasal cell behaviors, identifying two subpopulations within 4 weeks: RFP-only cells (basal cell-derived, never proliferated) and GFP^+^RFP^+^ cells (proliferated basal cell-derived) (Fig. 1f, g and Supplementary Fig. S3f, g). This supports the existence of at least two distinct basal subpopulations: one that proliferates to generate either basal cells or luminal cells, and another that differentiates directly into luminal cells. Although our current genetic tools preclude complete quantitative dissection, we provide substantial evidence supporting their existence.

Given regional differences in basal cell turnover, we next investigated whether this heterogeneity stems from divergent expansion properties. We developed a cell proliferation-induced clonal analysis system using a dual recombinase-mediated multi-colored reporter line: *R26-rox-Stop-rox-Confetti* (*R26-Confetti2*), activated by basal cell-specific Cre and proliferation-motivated Dre after Tam induction. This enables expression of a single confetti reporter (CFP, RFP, YFP, or GFP) in proliferating basal cells and their progeny (Supplementary Fig. S4a). Whole-mount imaging of tracheae from *P63-CreER*;*Ki67-L-Dre*;*R26-Confetti2* mice at 1, 2, and 4 weeks post-Tam induction showed a progressive increase in reporter^+^ cells predominantly restricted to dorsal regions without leakiness (Supplementary Fig. S4b–e). Tracheal sections at 4 weeks revealed multi-colored clones in both dorsal and ventral regions, confirming basal cell expansion (Fig. 1h). Quantitative assessment demonstrated striking regional differences, with dorsal enrichment of larger clones (Fig. 1i–k). Lower Tam dose yields fewer, smaller clones, mostly in dorsal regions (Supplementary Fig. S4f–i). Sequential sectioning of an RFP clone containing a basal cell and a luminal cell demonstrated that a single basal cell can proliferate to generate both lineages, consistent with R-RL-GFP reporter findings (Supplementary Fig. S2g, j). The above data indicate that dorsal basal cells exhibit superior proliferative expansion during homeostasis compared to ventral counterparts.

To assess regional heterogeneity during injury, we treated mice with naphthalene (NA) immediately after Tam. Three weeks post-injury, whole-mount imaging showed widespread GFP signals throughout dorsal and ventral regions (Supplementary Fig. S4k), indicating injury-induced activation of basal cells. Sirius Red staining and immunostaining of Collagen III and GFP revealed fibrosis and abundant GFP signals in both regions (Supplementary Fig. S4l, m). In *P63-CreER*;*Ki67-L-Dre*;*R26-Confetti2* mice, confetti signals were widespread post NA (Supplementary Fig. S4n). Immunostaining revealed distinct regional responses: while dorsal reporter^+^ cells maintained basal identity (KRT5^+^), ventral clones contained luminal cells expressing secretory (CC10) and ciliated (A-Tub) markers (Supplementary Fig. S4o). Quantitative analysis revealed significantly more labeled basal cells in the dorsal region post-NA versus sham, and more reporter^+^ secretory cells in the ventral regions (Supplementary Fig. S4p, q). Post-injury, homeostatic differences in clone size distribution were eliminated (Supplementary Fig. S4r, s). Similar systemic response throughout the trachea occurred after polidocanol (PDOC) injury (Supplementary Fig. S5). Our clonal analysis demonstrates that dorsal basal cells exhibit superior proliferative expansion during homeostasis, whereas ventral basal cells show greater potential to expand and differentiate into the luminal lineage during injury repair.

The Wnt/β-catenin pathway is crucial for embryonic development, stem cell maintenance, and tissue regeneration^15^. To investigate its role in tracheal epithelium, we performed scRNA-seq on dorsal and ventral tracheal tissues harvested from control and *P63-CreER;β-catenin*^*fl/fl*^ mice 10 days post-Tam. Clustering analysis identified major cell populations, including basal cells, secretory cells, and ciliated cells, alongside non-epithelial types (Fig. 1l; Supplementary Fig. S6a). Wnt signaling activity was significantly reduced in knockout (KO) epithelial cells (Supplementary Fig. S6b, c). Cell composition analysis revealed marked shifts across regions and genotypes (Fig. 1m). Notably, cell cycling-related gene expression was higher in the dorsal basal cells than ventral and decreased starkly in both KO basal cell populations (Fig. 1n). Pseudotime analysis delineated a trajectory from basal cells through secretory to ciliated cells (Fig. 1o). In the dorsal trachea, β-catenin deficiency led to an accumulation at the secretory stage and reduction at the ciliated stage, however, consistent with a ciliated-to-secretory lineage bias; these changes were less pronounced ventrally (Supplementary Fig. S6d, e).

Given that the stromal cells are often primary Wnt sources, we utilized CellChat to analyze cell–cell communication. While Wnt signaling strength remained stable in smooth muscle cells (SMCs) and fibroblasts, we detected a sharp decline in signaling from basal cells toward themselves and ciliated cells (Supplementary Fig. S6f). Interestingly, *Wnt4* was highly expressed in basal and ciliated cells rather than in non-epithelial populations. In controls, basal cells functioned as primary Wnt senders, a role that was nearly abolished in the *β-catenin*^*fl/fl*^ group (Supplementary Fig. S6g, h). Collectively, these data suggest tracheal epithelial cells, particularly basal cells, utilize autocrine and paracrine Wnt signaling to maintain homeostatic cell ratios.

To define β-catenin’s role in in situ basal cell proliferation, we integrated conditional knockout with proliferation tracing using the BC-fProTracer system. *BC-fProTracer;β-catenin*^*fl/fl*^ mice allowed simultaneous proliferation recording and *β-catenin* KO specifically in basal cells upon Tam induction (Fig. 1p; Supplementary Fig. S7a). Immunostaining at 4 weeks revealed that β-catenin loss significantly impaired dorsal basal cell proliferation while ventral proliferation remained unaffected (Fig. 1q, r). KRT13^+^ hillock cells were reduced after KO (Supplementary Fig. S7b), but their remaining proliferation capacity was unchanged (Supplementary Fig. S7c, d). Therefore, *β-catenin* KO affected the number but not the remaining proliferative capacity of hillock cells. Additionally, *β-catenin* KO also altered basal cell fate, significantly increasing GFP-labeled luminal cells derived from basal cells in both regions (Fig. 1q, r). These labeled luminal cells were predominantly secretory cells (Supplementary Fig. S7e–h). Our findings demonstrated that Wnt signaling played a dual role in tracheal basal cells: it regulates the symmetrical self-renewal of a subset of dorsal basal cells during homeostasis, and also constrains basal cell division and differentiation toward luminal cells in basal cells in both dorsal and ventral sides (Fig. 1s).

The regional proliferative difference in basal cells was consistently observed across three complementary experimental approaches: ubiquitous ProTracer recording, basal cell-specific BC-fProTracer tracing, and confetti-based clonal analysis. These methods revealed that basal cell proliferation during homeostasis primarily occurs through symmetric self-renewal to maintain the stem cell pool. Combining lineage and proliferation tracing of basal cells, we identified the two subpopulations (multipotent stem cells for self-renewal and basal luminal progenitors for differentiation) in both the ventral and dorsal regions. Spatial analysis refined this view: while basal luminal progenitors appear uniformly distributed based on the differentiation marker, multipotent stem cells show significant dorsal enrichment, evidenced by the dorsal proliferation hotspot where they undergo frequent symmetric divisions. Our findings also refined the perspective that hillock cells are the sole epithelial cell source for tracheal homeostasis and injury repair, revealing that while they are highly proliferative, other basal cells in the dorsal region also retain proliferative capacity for further supporting the observed dorsal-ventral proliferation difference. Furthermore, we identified functional heterogeneity in Wnt signaling among basal cells. Our results suggest β-catenin plays an important role in maintaining symmetrical self-renewal in dorsal basal stem cells while simultaneously restraining differentiation potential by limiting their differentiation towards luminal fates.

The dorsal enrichment of highly proliferative basal cells likely stems from region-specific niche signals. Distinct microenvironments created by dorsal smooth muscle and ventral cartilage may provide differential signaling cues. Additionally, dorsal and ventral basal cells experience different mechanical forces during tracheal smooth muscle contraction and relaxation cycles, which may underlie their divergent proliferative behavior. Further investigation is needed to define the specific niche factors from smooth muscle or regionally distinct fibroblasts that establish and maintain this proliferative hierarchy, and to investigate how regional heterogeneity influences responses to injury, infection, or diseases.

## Supplementary information

Suppmentary Figures and methods
