# Supplementary material for: Highly regional generation and heterogeneous differentiation of basal cells in mouse trachea
Source: Cell Discov. 2026 Mar 30;12:22. doi: 10.1038/s41421-026-00887-4 (PMC13035939; doi:10.1038/s41421-026-00887-4)
Supplement: Supplementary file 1 — Suppmentary Figures and methods [file 41421_2026_887_MOESM1_ESM.pdf]

Supplementary Information for

Highly regional generation and heterogeneous differentiation of basal cells in mouse trachea

Xiuxiu Liu[1,4], Wendong Weng[1,4],Zhen He[1,4] , Kuo Liu[2], Maoying Han[1], Wenjuan Pu1,*, Bin Zhou[1-3]

[1]CAS CEMCS-CUHK Joint Laboratory, New Cornerstone Science Laboratory, State Key Laboratory of Cell Biology, Shanghai Institute of Biochemistry and Cell Biology, Center for Excellence in Molecular Cell Science, Chinese Academy of Sciences, University of Chinese Academy of Sciences, Shanghai, China

[2]Key Laboratory of Systems Health Science of Zhejiang Province, School of Life Science, Hangzhou Institute for Advanced Study, University of Chinese Academy of Sciences, Hangzhou, China

[3]School of Life Science and Technology, ShanghaiTech University, Shanghai, China

[4]These authors contributed equally to this work.

Correspondence: puwenjuan@sibcb.ac.cn (W.P.) and zhoubin@sibs.ac.cn (B.Z.)

This PDF file includes:

Materials and Methods

Acknowledgements

Conflict of interest

Supplementary information, Figs. S1-S7

Materials and Methods

Mice

All mouse experiments were conducted in accordance with the current guidelines of the Institutional Animal Care and Use Committee (IACUC) at the Center for Excellence in Molecular Cell Science, Shanghai Institute of Biochemistry and Cell Biology, Chinese Academy of Sciences. All mice were maintained on a 129/C57BL6 and ICR-mixed background and were housed in a Specific Pathogen Free (SPF) facility with individually ventilated cages. The room has controlled temperature (20–25 °C), humidity (30–70%), and light (12 hours light-dark cycle). The mouse lines *R26-DreER*, *Ki67-CrexER*, *R26-L-GFP*, *P63-CreER*, *R26-tdT*, *Ki67-L-Dre*, *R26-RL-GFP*, *R26-Confetti2*, *β-catenin*$^{fl/+}$ were previously reported [1-3]. For CreER/DreER recombination induction, tamoxifen (Sigma, T5648) was dissolved in corn oil (20 mg/mL) and administered via oral gavage at the indicated time points in the dose of 0.2 mg/g of body weight.

Wholemount staining of trachea

Trachea tissues were stained as previously described [4]. Mouse tracheae were collected, and the luminal surface was exposed by making a vertical incision along the anterior wall. The tissues were fixed in 4% paraformaldehyde for 20 minutes at room temperature (RT), followed by three PBS washes for 1 h in total. Next, tissues were permeabilized with PBS containing 0.1% Tween for 15 minutes at room temperature. For immunostaining, tissues were incubated overnight at 4°C with primary antibodies diluted in PBS with 0.2% Triton X-100 (0.2% PBST) under gentle inversion. After washing in PBS for 1 hour, tissues were incubated with secondary antibodies diluted in 0.2% PBST for 3 hours at 4°C. Finally, tissues were washed in PBS for 1 h and mounted for imaging.

Tissue collection and immunofluorescent staining

Immunostaining was performed as described [5]. Briefly, tissues were collected in cold PBS, subsequently fixed in 4% PFA at 4 °C for 1 hour, washed 3 times in cold PBS, dehydrated in 30% sucrose/PBS overnight at 4 °C, then embedded in OCT compound (Sakura) and stored at -80°C for cryosection. Cryosections of 10 μm thickness were placed on positively charged slides and stored at -20 °C until use. For immunostaining, tissue sections were blocked with PBSST (0.2% Triton X-100, 2.5% normal donkey serum in PBS) for 30 minutes at room temperature, subsequently incubated overnight with primary antibodies at 4 °C. The next day, tissue sections were washed three times with PBS and incubated with Alexa fluorescence-conjugated secondary antibodies (Invitrogen) for 30 minutes at room temperature, then washed three more times with PBS and mounted with mounting medium. HRP-conjugated antibodies with tyramide signal amplification kit were used to amplify weak signals. GS-IB4, Alexa Fluor™ 647 (Thermo Fisher, I32450; diluted 1:300) was used to stain for tracheal basal cells. The primary antibodies used were: tdTomato (Rockland, 600-401-379; diluted 1:1000), GFP (nacalai tesque, 04404-84; diluted 1:500), GFP (Rockland, 600-101-215; diluted 1:500), Keratin 5 (Krt5) (Biolegend, 905504; diluted 1:500), Keratin 8 (Krt8) (DSHB, Troma-I-C; diluted 1:500), Keratin 13 (Krt13) (Abcam,

ab92551; diluted 1:500), Acetylated Tubulin (Sigma, T7451; diluted 1:500), Uteroglobin (CC10) (Abcam, ab213203; diluted 1:300), β-catenin (BD Pharmingenp, 610153; diluted 1:200), PDGFRa (R&D, AF1062; diluted 1:200), CD45 (eBioscience, 17-0451-82; diluted 1:400), VE-cadherin (R&D, AF1002; diluted 1:100). The secondary antibodies used were: Alexa donkey a-rat 488 (Invitrogen, A21208; diluted 1:1000), Alexa donkey a-goat 488 (Invitrogen, A11055; diluted 1:1000), Alexa donkey a-rabbit 555 (Invitrogen, A31572; diluted 1:1000), Alexa donkey a-mouse 555 (Invitrogen, A31570; diluted 1:1000), Alexa donkey a-goat 555 (Invitrogen, A21432; diluted 1:1000), Alexa donkey a-rat 647 (Invitrogen, A48272; diluted 1:1000), Alexa donkey a-mouse 647 (Invitrogen, A31571; diluted 1:1000), Alexa donkey a-goat 647 (Invitrogen, A21447; diluted 1:1000), HRP-donkey-a-rat (JIR, 712-035-153, 1:100). Images were acquired using an Olympus FV4000 confocal microscope (Evident FV4000) and processed with the FIJI/ImageJ and Photoline software.

Paraffin embedding and immunofluorescent staining

The tissue was fixed using 4% PFA at 4 °C for 12 hours and placed in the embedding box. Then the tissue was soaked in sequence with 75% ethanol for 1.5 hours twice, 85% ethanol for 1.5 hours, 90% ethanol for 1.5 hours, 95% ethanol for 1.5 hours, anhydrous ethanol for 1 hour twice, anhydrous ethanol for 1.5 hours, anhydrous ethanol with xylene (1:1) for 30 minutes, xylene for 30 minutes twice, and embedded in paraffin wax. Paraffin-embedded tissue was cut into 3 μm sections, flattened in 42 °C water, and lifted with slides. The slides were put into a 65 °C oven to bake until the water dries up and the paraffin melts. Then the slides were de-paraffined by soaking in sequence with xylene for 5 minutes twice, anhydrous ethanol for 2 minutes twice, 95% ethanol for 2 minutes, 75% ethanol for 2 minutes, and washed with water. For antigen retrieval, tissue sections were heated in citrate solution (pH 6.0) or EDTA solution (pH 9.0) for 15 minutes, then washed three times with PBS. To block endogenous peroxidase, tissue sections were incubated in 3% hydrogen peroxide solution for 25 minutes at room temperature, then washed three times with PBS. For immunostaining, tissue sections were blocked with 3% BSA for 10 minutes at room temperature, subsequently incubated overnight with the primary antibody at 4 °C. The next day, tissue sections were washed three times with PBS and incubated with HRP-conjugated antibody for 1 hour at room temperature, then washed three times with PBS. Finally, the tissue sections were incubated with fluorescent chromogenic agent for 5 minutes and washed with PBS. For staining multiple antibodies on one tissue section, the process from antigen retrieval to chromogenic reaction needs to be repeated. Next, the tissue sections were incubated with DAPI for 10 minutes at room temperature, then washed three times with PBS and mounted with mounting medium. The primary antibodies used were: GFP (Rockland, 600-101-215; diluted 1:500), Foxj1 (Invitrogen, 14-9965-82; diluted 1:200), Notch3 (Cell Signaling, 5276T; diluted 1:50). The secondary antibodies used were: HRP-donkey-a-mouse (JIR, 715-035-150; diluted 1:100), HRP-donkey-a-rabbit (JIR, 711-035-152; diluted 1:100), Tyramide signal amplification kit (Abclonal, RK05903; diluted 1:1000). Images were acquired using an Olympus FV4000 confocal microscope (Evident FV4000) and processed with the FIJI/ImageJ and Photoline software.

Tissue collection and EdU staining

For EdU incorporation analysis, mice were injected intraperitoneally with EdU (Invitrogen, A10044) at the dose of 10 mg/kg body weight 24 hours before mice sacrifice. EdU incorporation was detected by using the Click-iT EdU Alexa Fluor 647 Imaging Kit (Invitrogen, C10340). Images were acquired using an Olympus confocal microscope (Evident FV4000). Images were analyzed by the ImageJ/FIJI and Photoline software.

Naphthalene and Polidocanol injury

The tracheal injury model induced by naphthalene was carried out as previously described [6,7]. Naphthalene (Sigma 84679) was freshly dissolved in sterile corn oil at a concentration of 25 mg/ml. To trace the proliferation and clonal expansion after airway injury, naphthalene was administered intraperitoneally to the mice at a dose of 25 μg/g of body weight. For polidocanol injury, 20 μL 2% polidocanol (wt/vol) was delivered intratracheally through the tongue-pull method as previously reported[8]. Trachea tissues were collected 3 weeks after injuries for clonal analysis.

Sing-cell RNA sequencing

Tracheal tissues were dissociated into single-cell suspensions using a papain-based enzymatic digestion protocol (Worthington) supplemented with DNase I, followed by mechanical trituration and quenching with ovo-mucoid protease inhibitor. Single-cell libraries were constructed using the Chromium GEM-X Single Cell 5′ v3 platform (10x Genomics). For bioinformatic processing, ambient RNA was removed using SoupX (v1.6.2), and doublets were excluded via scDblFinder (v1.12.0). Data integration across dorsal/ventral and control/KO groups was performed in Seurat (v5.2.1) using SCTransform normalization and Canonical Correlation Analysis (CCA). To model epithelial differentiation, developmental trajectories were inferred using monocle3 (v1.3.1), with basal cells defined as the root. Intercellular signaling dynamics were analyzed using CellChat (v2.1.2) to quantify Wnt-specific communication, while pathway activity was assessed using Seurat's AddModuleScore function based on downstream target genes.

Statistical analysis

All of the data were obtained from at least 3 individual mouse samples and presented as mean values ± SEM. An unpaired Student's t-test was performed for statistical data comparison between dorsal and ventral or *β-catenin* knockout and control. $P \leqq 0.05$ indicated statistical significance; differences were considered as not statistically significant when $P > 0.05$.

References

1 He, L. *et al.* Proliferation tracing reveals regional hepatocyte generation in liver homeostasis and repair. *Science* **371** (2021). https://doi.org:10.1126/science.abc4346

2 Liu, X. *et al.* Functional ProTracer identifies patterns of cell proliferation in tissues and underlying regulatory mechanisms. *NPJ Regen Med* **8**, 41 (2023). https://doi.org:10.1038/s41536-023-00318-y
3 Han, X. *et al.* Lineage Tracing Reveals the Bipotency of SOX9(+) Hepatocytes during Liver Regeneration. *Stem Cell Reports* **12**, 624-638 (2019). https://doi.org:10.1016/j.stemcr.2019.01.010
4 Lin, B. *et al.* Airway hillocks are injury-resistant reservoirs of unique plastic stem cells. *Nature* (2024). https://doi.org:10.1038/s41586-024-07377-1
5 Liu, X. *et al.* Cell proliferation fate mapping reveals regional cardiomyocyte cell-cycle activity in subendocardial muscle of left ventricle. *Nat Commun* **12**, 5784 (2021). https://doi.org:10.1038/s41467-021-25933-5
6 Liu, K. *et al.* Tracing the origin of alveolar stem cells in lung repair and regeneration. *Cell* **187**, 2428-2445.e2420 (2024). https://doi.org:10.1016/j.cell.2024.03.010
7 Liu, Q. *et al.* Lung regeneration by multipotent stem cells residing at the bronchioalveolar-duct junction. *Nat Genet* **51**, 728-738 (2019). https://doi.org:10.1038/s41588-019-0346-6
8 Ma, L. *et al.* Life-long functional regeneration of in vivo airway epithelium by the engraftment of airway basal stem cells. *Nat Protoc* **20**, 810-842 (2025). https://doi.org:10.1038/s41596-024-01067-y

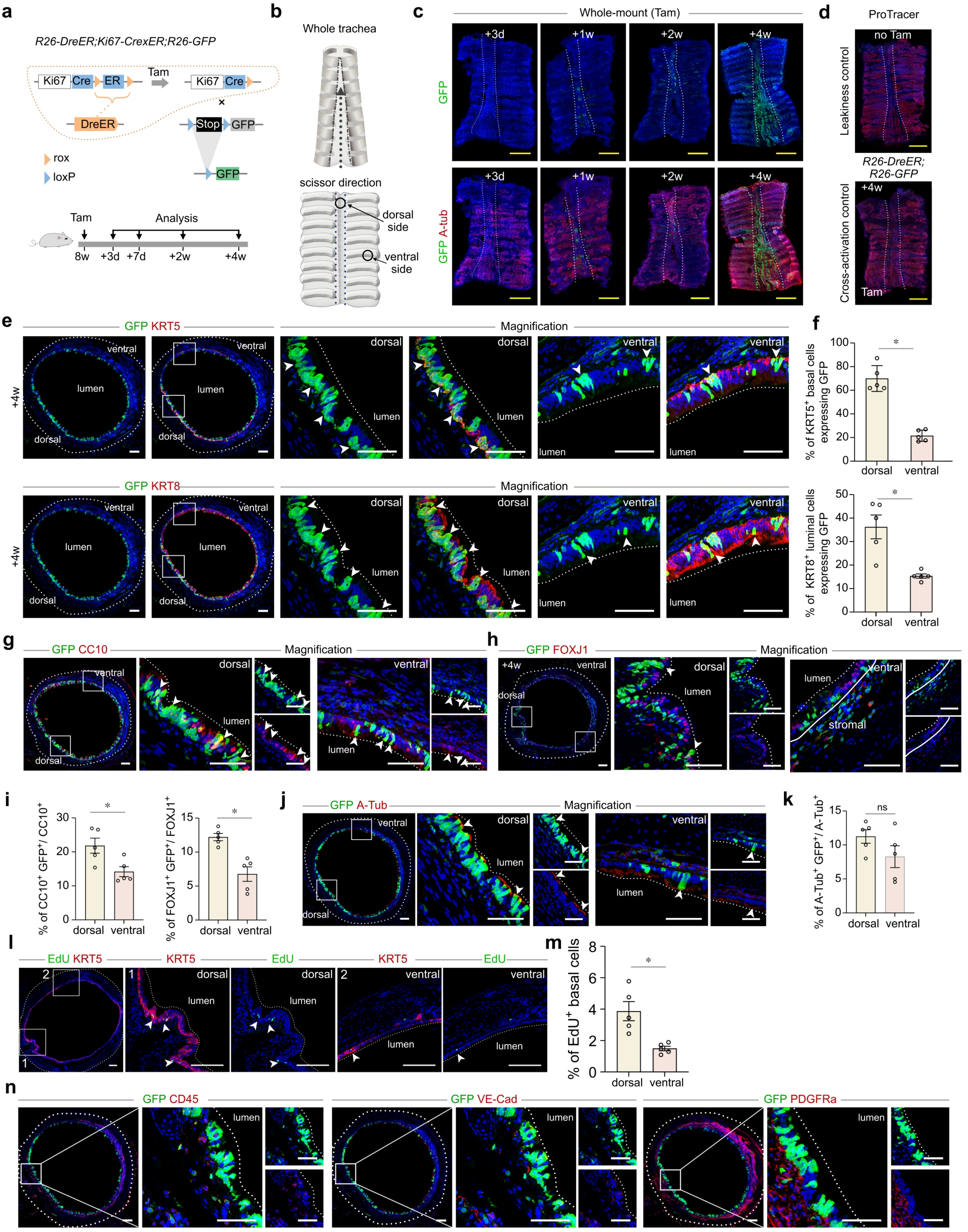

a
R26-DreER;Ki67-CrexER;R26-GFP
Ki67 Cre ER
Tam
Ki67 Cre
DreER
Stop GFP
GFP
rox
loxP
Tam
Analysis
8w +3d +7d +2w +4w
b
Whole trachea
scissor direction
dorsal side
ventral side
c
Whole-mount (Tam)
+3d +1w +2w +4w
GFP
GFP A-tub
d
ProTracer
no Tam
Leakiness control
R26-DreER; R26-GFP
+4w
Cross-activation control
Tam
e
GFP KRT5
Magnification
+4w
ventral
lumen
dorsal
GFP KRT8
f
% of KRT5+ basal cells expressing GFP
% of KRT8+ luminal cells expressing GFP
dorsal ventral
g
GFP CC10
h
GFP FOXJ1
stromal
i
% of CC10+ GFP+/ CC10+
% of FOXJ1+ GFP+/ FOXJ1+
j
GFP A-Tub
k
% of A-Tub+ GFP+/A-Tub+
ns
l
EdU KRT5
KRT5
EdU
m
% of EdU+ basal cells
n
GFP CD45
GFP VE-Cad
GFP PDGFRa

Supplementary Figure 1: Basal cell proliferation captured by the ProTracer system.

a Schematic of the ProTracer experimental design for recording tracheal epithelial cell proliferation. **b** Schematic demonstrating trachea dissection by ventral incision to expose the luminal surface and orientation for whole-mount imaging. Ventral incision exposes the dorsal surface. **c** Whole-mount immunofluorescence staining of acetyl-tubulin (A-Tub) and GFP on trachea tissues was analyzed at different time points post-tamoxifen (Tam) induction. Dotted line demarcates dorsal region. **d** Control experiments were performed as corn oil-treated (no Tam) ProTracer mice or Tam-treated R26-DreER;R26-GFP mice. The dotted line demarcates the dorsal region. **e** Cross-trachea sections from *R26-DreER;Ki67-CrexER;R26-GFP* mice 4 weeks post-Tam, stained for GFP with basal cell marker KRT5 or luminal cell marker KRT8. **f** Quantification of GFP^+ basal cell ($KRT5^+$) or luminal cells ($KRT8^+$) in dorsal versus ventral regions. **g**, **h** Trachea sections from 4 weeks post-Tam stained for GFP with secretory cell marker CC10 (**g**) or ciliated cell marker FOXJ1 (**h**). **i** Quantification of GFP^+ secretory cells and ciliated cells in dorsal versus ventral regions. **j** Trachea sections from 4 weeks post-Tam stained for GFP with ciliated cell marker A-tub. Arrowheads indicate the GFP^+A-tub^+ cells. **k** Quantification of GFP^+ A-Tub^+ ciliated cells in dorsal versus ventral regions. **l** EdU labeling (24-hour pulse) in wild-type mice. Trachea sections stained for EdU and KRT5. **m** Quantification of the EdU^+ basal cells confirms regional proliferation bias. An unpaired t-test was used to analyze differences between groups. **n** GFP co-staining with CD45 (immune cells), VE-Cad (endothelial cells), and Pdgfra (fibroblasts) 4 weeks post-Tam. ✻$P \leqq 0.05$; ns (not significant), $P > 0.05$. Data represent mean ± SEM. Scale bars: yellow, 1000 μm; white, 100 μm.

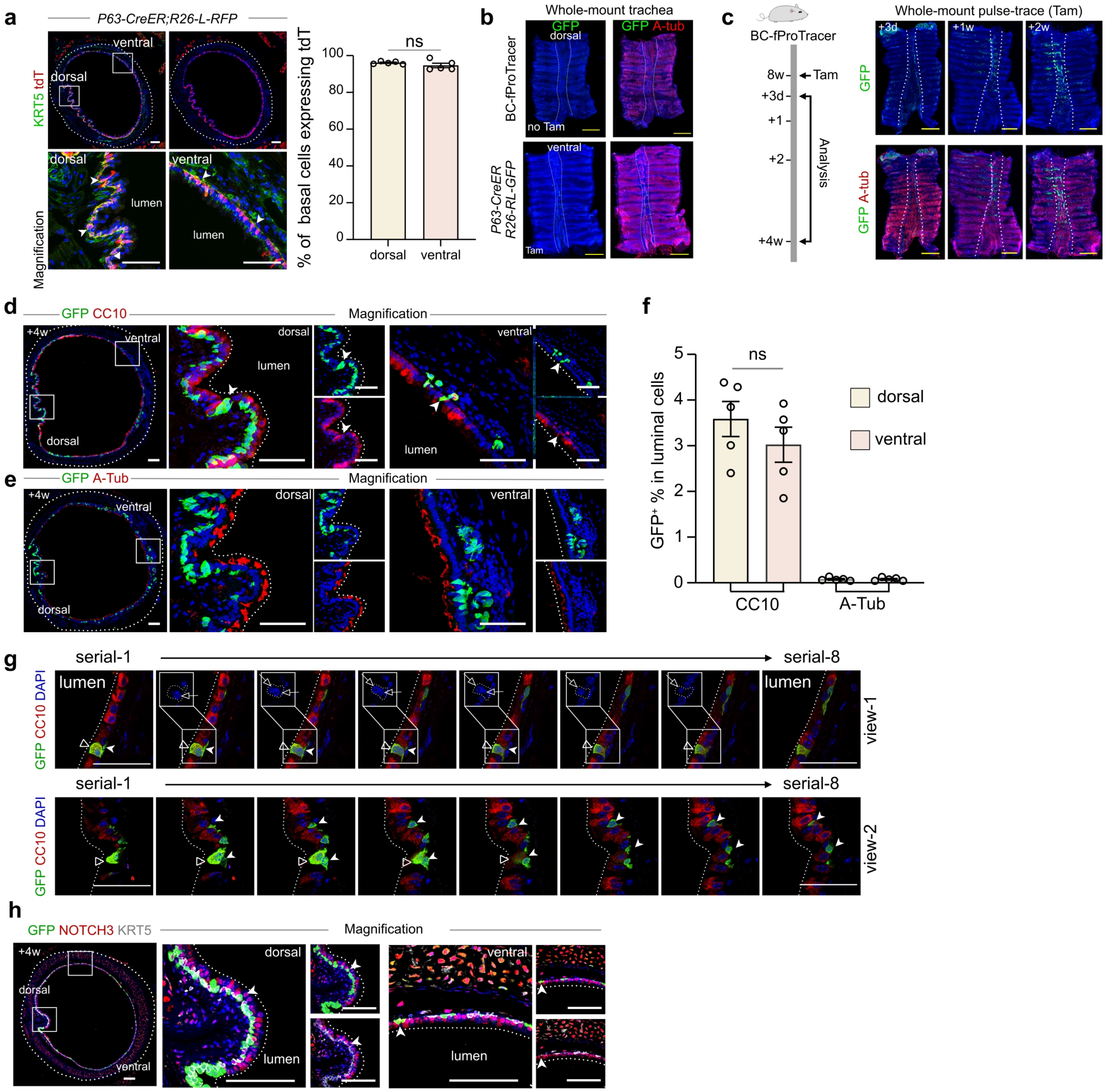

a
P63-CreER;R26-L-RFP
KRT5 tdT
dorsal
ventral
lumen
Magnification
% of basal cells expressing tdT
ns
100
80
60
40
20
0
dorsal
ventral
b
Whole-mount trachea
GFP
GFP A-tub
dorsal
ventral
no Tam
Tam
BC-fProTracer
P63-CreER R26-RL-GFP
c
BC-fProTracer
8w
Tam
+3d
+1
+2
+4w
Analysis
Whole-mount pulse-trace (Tam)
+3d
+1w
+2w
GFP
GFP A-tub
d
GFP CC10
Magnification
+4w
ventral
dorsal
lumen
e
GFP A-Tub
Magnification
+4w
ventral
dorsal
f
GFP⁺ % in luminal cells
5
4
3
2
1
0
ns
CC10
A-Tub
dorsal
ventral
g
serial-1
serial-8
GFP CC10 DAPI
lumen
view-1
view-2
h
GFP NOTCH3 KRT5
Magnification
+4w
dorsal
ventral
lumen

Supplementary Figure 2: Heterogeneity of basal cell proliferation revealed by BC-fProTracer

a Validation of *P63-CreER* specificity by staining for tdTomato in basal cells ($KRT5^+$) post-Tam. **b** Negligible GFP in corn oil-treated (no Tam) BC-fProTracer or Tam-treated *P63-CreER;R26-RL-GFP* mice. **c** Whole-mount staining (A-Tub, GFP) in BC-fProTracer mice at different time points post-Tam. **d**, **e** Sections stained for GFP with CC10 (secretory cells) or A-Tub (ciliated cells). **f** Quantification of the GFP^+ secretory and ciliated cells derived from basal cell proliferation. **g** Serial pictures of GFP^+ cells revealed one club cell derived from proliferated basal cells. **h** GFP^+ basal cells were analyzed by co-staining with NOTCH3. Arrowheads indicate the $GFP^+NOTCH3^+$ cells. Statistical analysis: Unpaired t-test; ✻ $P \leqq 0.05$; ns, $P > 0.05$. Data represent mean ± SEM. Scale bars: yellow, 1000 µm; white, 100 µm.

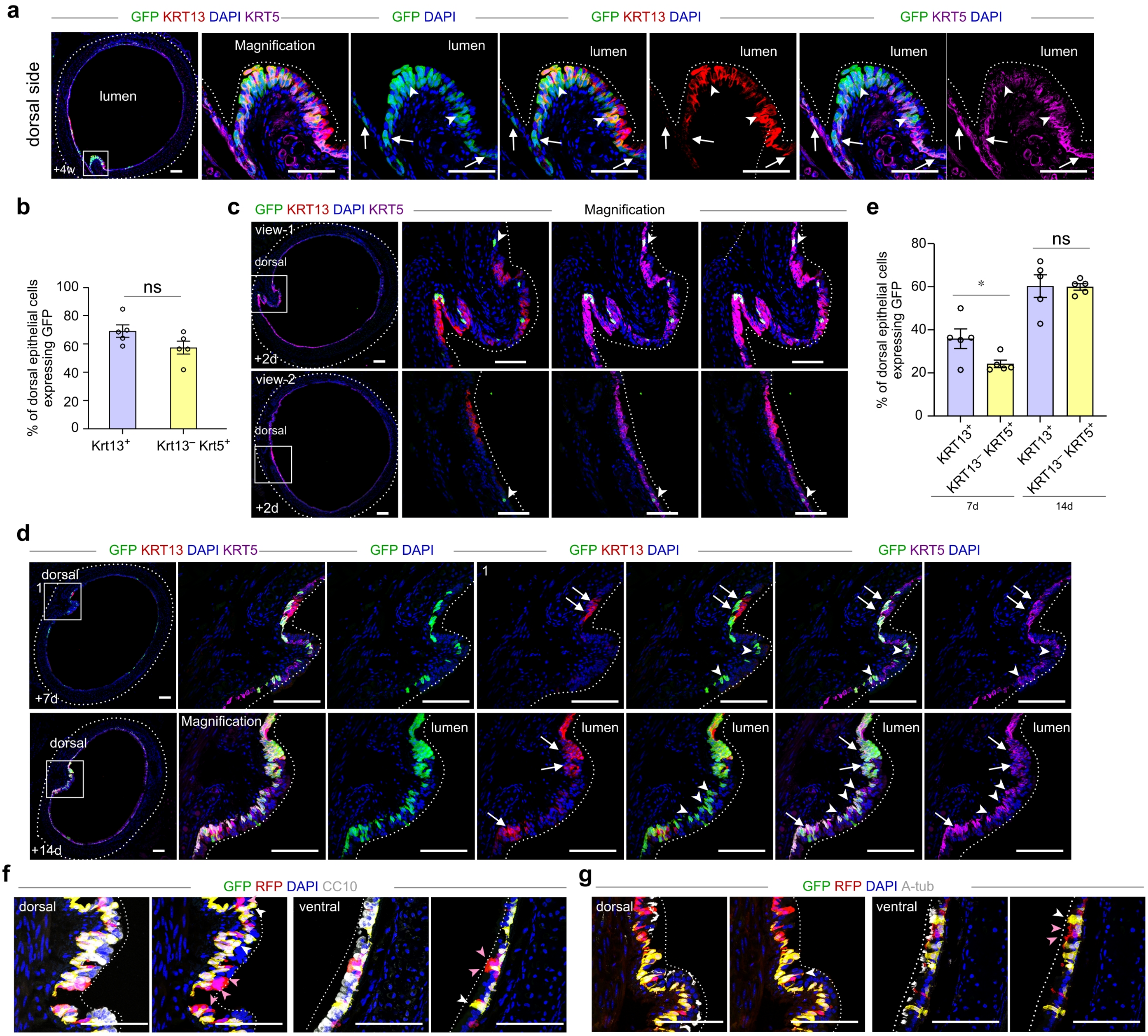

a
GFP KRT13 DAPI KRT5
GFP DAPI
GFP KRT13 DAPI
GFP KRT5 DAPI
dorsal side
lumen
Magnification
+4w
b
% of dorsal epithelial cells expressing GFP
100
80
60
40
20
0
ns
Krt13+
Krt13− Krt5+
c
GFP KRT13 DAPI KRT5
Magnification
view-1
view-2
dorsal
+2d
e
% of dorsal epithelial cells expressing GFP
80
60
40
20
0
*
ns
KRT13+
KRT13− KRT5+
7d
14d
d
GFP KRT13 DAPI KRT5
GFP DAPI
GFP KRT13 DAPI
GFP KRT5 DAPI
dorsal
1
+7d
+14d
Magnification
lumen
f
GFP RFP DAPI CC10
dorsal
ventral
g
GFP RFP DAPI A-tub
dorsal
ventral

Supplementary Figure 3: Proliferated basal cells versus hillock cells

a Co-staining for GFP, KRT13 (hillock), and KRT5 (basal) on trachea sections collected from mice 4 weeks post Tam. **b** Quantification of GFP labeling efficiency in $KRT13^{+}$ vs. $KRT13^{-}$ basal subpopulations (dorsal). **c** Co-staining for GFP, KRT13 (hillock), and KRT5 (basal) on trachea sections collected from mice 2 days post Tam. Arrows indicate $GFP^{+}KRT13^{+}$ proliferating hillock cells, while arrowheads indicate proliferating non-hillock basal cells. **d** Trachea sections from 7 days or 14 days post-Tam were stained with GFP, KRT13, and KRT5. Arrows indicate $GFP^{+}KRT13^{+}$ proliferating hillock cells, while arrowheads indicate proliferating non-hillock basal cells. **e** Quantification of GFP labeling efficiency in $KRT13^{+}$ vs. $KRT13^{-}$ basal subpopulations (dorsal). **f**, **g** Cross-trachea sections collected 4 weeks post-Tam from *P63-CreER;Ki67-L-Dre;R RFP/RL-GFP* mice were stained with CC10 (**f**, secretory cell) and A-Tub (**g**, ciliated cell). White arrowheads indicate $GFP^{+}RFP^{+}$ cells, while red arrowheads indicate RFP^{+} cells. Statistical analysis: Unpaired t-test; ✻ $P \leqq 0.05$; ns, $P > 0.05$. Data represent mean ± SEM. Scale bars: 100 μm.

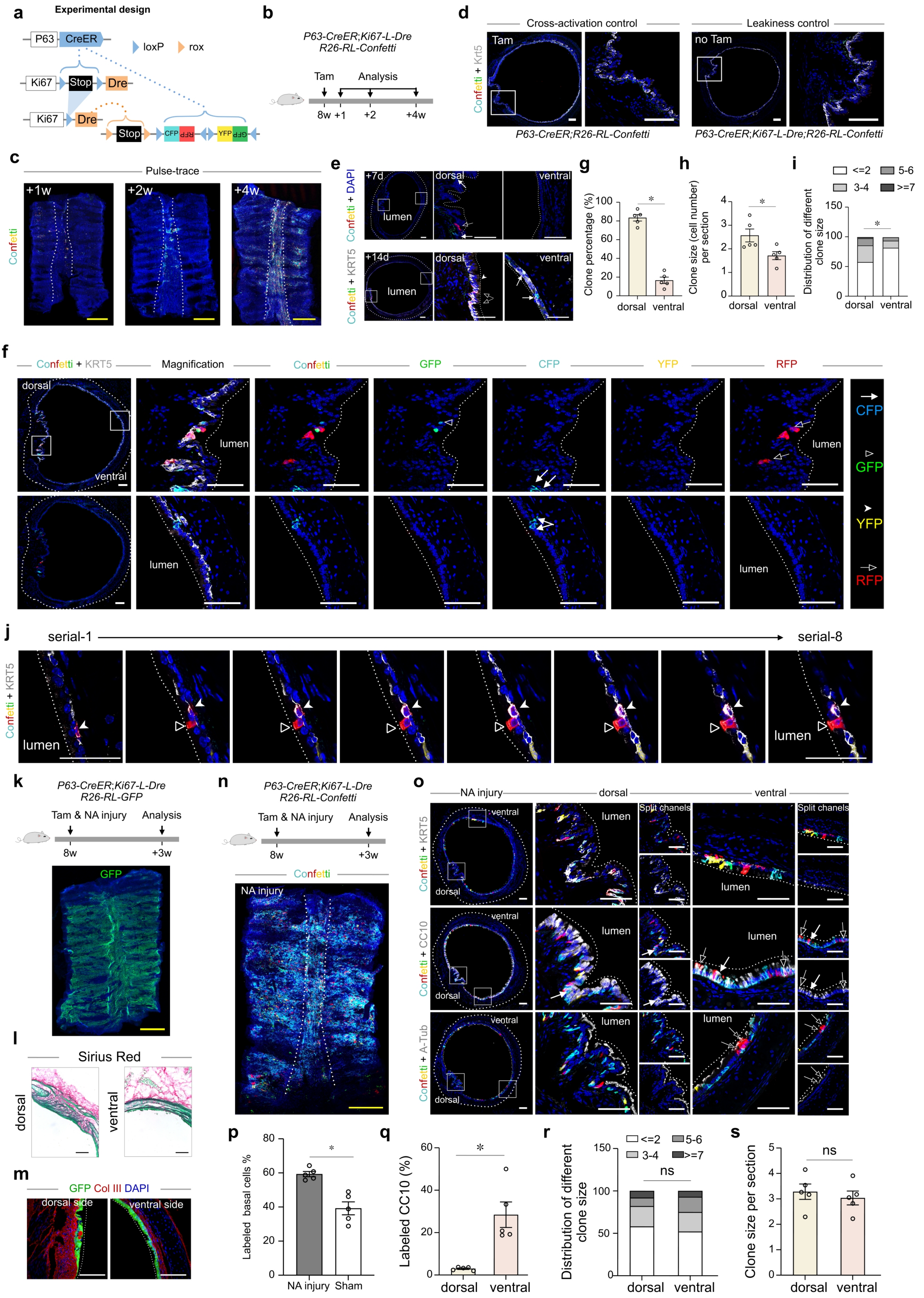

a
Experimental design
P63 CreER
loxP
rox
Ki67 Stop Dre
Ki67 Dre Stop CFP RFP YFP GFP
b
P63-CreER;Ki67-L-Dre
R26-RL-Confetti
Tam
Analysis
8w +1 +2 +4w
d
Cross-activation control
Leakiness control
Confetti + Krt5
Tam
no Tam
P63-CreER;R26-RL-Confetti
P63-CreER;Ki67-L-Dre;R26-RL-Confetti
c
Pulse-trace
Confetti
+1w
+2w
+4w
e
Confetti + DAPI
Confetti + KRT5
+7d
+14d
lumen
dorsal
ventral
g
Clone percentage (%)
dorsal ventral
h
Clone size (cell number) per section
dorsal ventral
i
<=2
5-6
3-4
>=7
Distribution of different clone size
dorsal ventral
f
Confetti + KRT5
Magnification
Confetti
GFP
CFP
YFP
RFP
dorsal
ventral
lumen
j
serial-1
serial-8
Confetti + KRT5
lumen
k
P63-CreER;Ki67-L-Dre
R26-RL-GFP
Tam & NA injury
Analysis
8w +3w
GFP
n
P63-CreER;Ki67-L-Dre
R26-RL-Confetti
Tam & NA injury
Analysis
8w +3w
Confetti
NA injury
o
NA injury
dorsal
ventral
Split chanels
Confetti + KRT5
Confetti + CC10
Confetti + A-Tub
lumen
l
Sirius Red
dorsal
ventral
m
GFP Col III DAPI
dorsal side
ventral side
p
Labeled basal cells %
NA injury Sham
q
Labeled CC10 (%)
dorsal ventral
r
<=2
5-6
3-4
>=7
ns
Distribution of different clone size
dorsal ventral
s
ns
Clone size per section
dorsal ventral

Supplementary Figure 4: Regional heterogeneity in basal cell proliferation revealed by clonal analysis.

a Schematic of the proliferation-dependent clonal analysis system (P63-CreER;Ki67-L-Dre;R26-confetti2). **b** The timeline of mouse tamoxifen induction and tissue analysis. **c** Whole-mount confetti images post-Tam show time-dependent clonal expansion primarily in dorsal regions (dotted line). **d** Controls: negligible confetti labeling in corn oil-treated (no Tam) mice or Tam-treated P63-CreER;R26-RL-Confetti mice (lacking Dre). **e** $Confetti^{+}$ cell clones after 7 and 14 days post-Tam. Arrow and arrowheads indicate split clones. **f** $Confetti^{+}$ cell clones 4 weeks after low dosage tam induction. Arrowheads indicate the GFP, CFP, and RFP clones. **g** Quantification of clone distribution (dorsal vs. ventral). **h** Quantification of average clone size. **i** Clone size distribution in dorsal vs. ventral regions, using Chi-square test. **j** Serial pictures of a RFP^{+} clone revealed one club cell and one basal cell. The hollow arrowheads indicate a luminal cell, while the arrowheads indicate a basal cell. **k** Whole-mount image of BC-fProTracer mouse trachea collected 3 weeks post-Tam and naphthalene (NA) injury. **l** Sirius Red staining of the trachea sections showed the fibrosis in both dorsal and ventral sides after NA injury. **m** Immunostaining of Col III and GFP on trachea sections after NA injury revealed the collagen deposition. **n** Whole-mount confetti signals 3 weeks post-Tam and NA injury. **o** Post-NA trachea sections were co-stained with confetti and KRT5 (basal), CC10 (secretory), and A-Tub (ciliated). **p** Quantification of the labeled basal cells by confetti after NA injury vs sham mouse trachea. **q** Quantification of $confetti^{+}$ secretory cells ($CC10^{+}$) post-NA. **r**, **s** Quantification of clone distribution (**r,** Chi-square test) and size (**s**) post-NA injury.

Statistical analysis: Unpaired t-test; ⁎ $P \leqq 0.05$; ns, $P > 0.05$. Data represent mean ± SEM. Scale bars: yellow, 1000 μm; white, 100 μm.

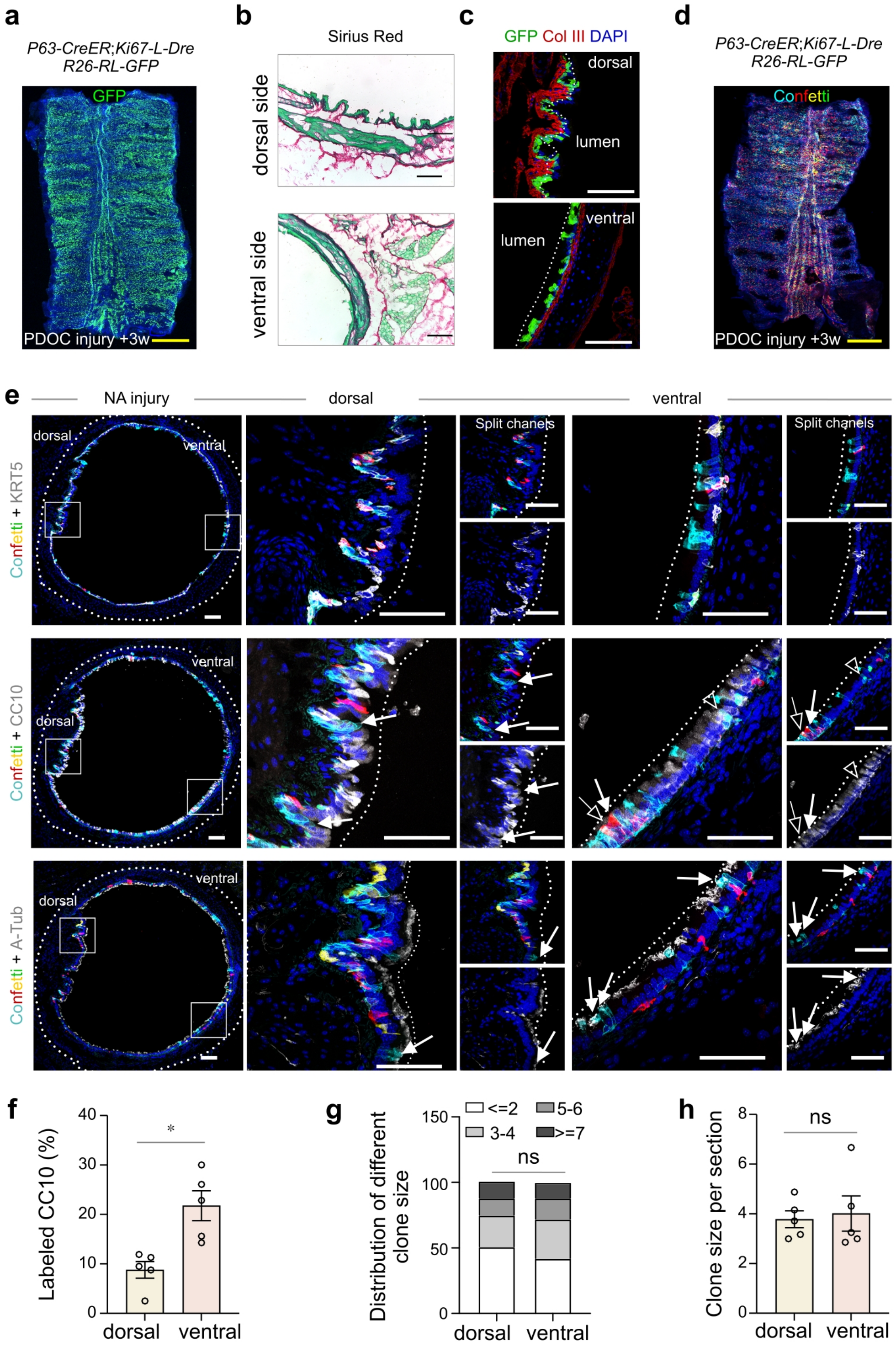

a
P63-CreER;Ki67-L-Dre
R26-RL-GFP
GFP
PDOC injury +3w
b
Sirius Red
dorsal side
ventral side
c
GFP Col III DAPI
dorsal
lumen
lumen
ventral
d
P63-CreER;Ki67-L-Dre
R26-RL-GFP
Confetti
PDOC injury +3w
e
NA injury
dorsal
ventral
Split chanels
Split chanels
Confetti + KRT5
Confetti + CC10
Confetti + A-Tub
dorsal
ventral
f
Labeled CC10 (%)
*
dorsal
ventral
g
Distribution of different clone size
<=2
5-6
3-4
>=7
ns
dorsal
ventral
h
Clone size per section
ns
dorsal
ventral

Supplementary Figure 5: Basal cell proliferation after trachea PDOC injuries.

a Whole-mount image of BC-fProTracer mouse trachea collected 3 weeks post-Tam and PDOC injury. **b** Sirius Red staining of the trachea after PDOC injury. **c** Col III staining on trachea sections after PDOC injury. **d** Whole-mount confetti images 3 weeks after PDOC injury. **e** Trachea sections collected 3 weeks post-PDOC injury were co-stained with confetti and KRT5 (basal), CC10 (secretory), and A-Tub (ciliated). Arrows and arrowheads indicate confetti^{+}marker^{+} cells. **f** Quantification of confetti^{+} secretory cells (CC10^{+}) post-PDOC injury. **g, h** Quantification of clone distribution (**g**, Chi-square test) and size (**h**) post-PDOC injury.

Statistical analysis: Unpaired t-test; ✻ $P \leqq 0.05$; ns, $P > 0.05$. Data represent mean ± SEM. Scale bars: yellow, 1000 µm; white, 100 µm.

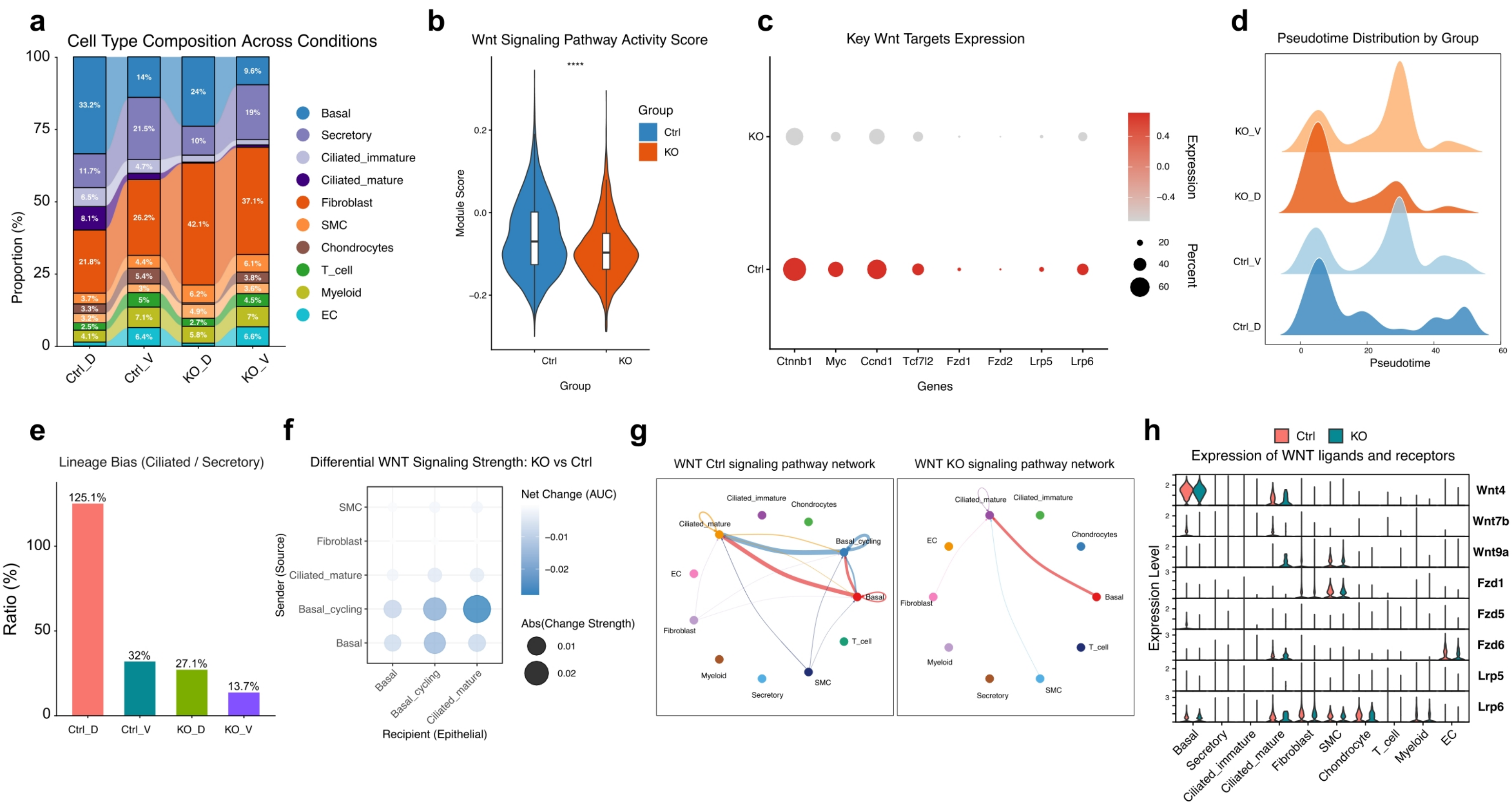
a
Cell Type Composition Across Conditions
Proportion (%)
Ctrl_D
Ctrl_V
KO_D
KO_V
Basal
Secretory
Ciliated_immature
Ciliated_mature
Fibroblast
SMC
Chondrocytes
T_cell
Myeloid
EC
b
Wnt Signaling Pathway Activity Score
Module Score
Group
Ctrl
KO
****
c
Key Wnt Targets Expression
Genes
Ctnnb1
Myc
Ccnd1
Tcf7l2
Fzd1
Fzd2
Lrp5
Lrp6
Expression
Percent
d
Pseudotime Distribution by Group
Pseudotime
e
Lineage Bias (Ciliated / Secretory)
Ratio (%)
125.1%
32%
27.1%
13.7%
f
Differential WNT Signaling Strength: KO vs Ctrl
Sender (Source)
Recipient (Epithelial)
Basal_cycling
Net Change (AUC)
Abs(Change Strength)
g
WNT Ctrl signaling pathway network
WNT KO signaling pathway network
h
Expression of WNT ligands and receptors
Expression Level
Wnt4
Wnt7b
Wnt9a
Fzd1
Fzd5
Fzd6
Lrp5
Lrp6
Chondrocyte

Supplementary Figure 6. Single-cell profiling of tracheal homeostasis and Wnt signaling networks in control and β-catenin$^{fl/fl}$ KO groups.

a scRNA-seq identification of cell populations. Stacked bar charts illustrate cell type composition across dorsal and ventral tracheal samples. **b**, **c** Evaluation of Wnt signaling activity in epithelial cells. (**b**) Violin plot showing the distribution of module scores. (**c**) Dot plot showing the expression of key Wnt targets and receptors. **d, e** Lineage trajectory and pseudotime analysis. (**d**) Ridge plot showing pseudotime distribution by group. (**e**) Lineage bias is calculated as the ratio of ciliated to secretory cells. **f-h** Cell-cell communication analysis of the Wnt pathway. (**f**) Bubble plot showing the net change in signaling strength (KO vs. Ctrl). (**g**) Circle plots of signaling networks in Ctrl and KO groups. (**h**) Violin plots showing expression levels of major Wnt ligands and receptors across all cell types.

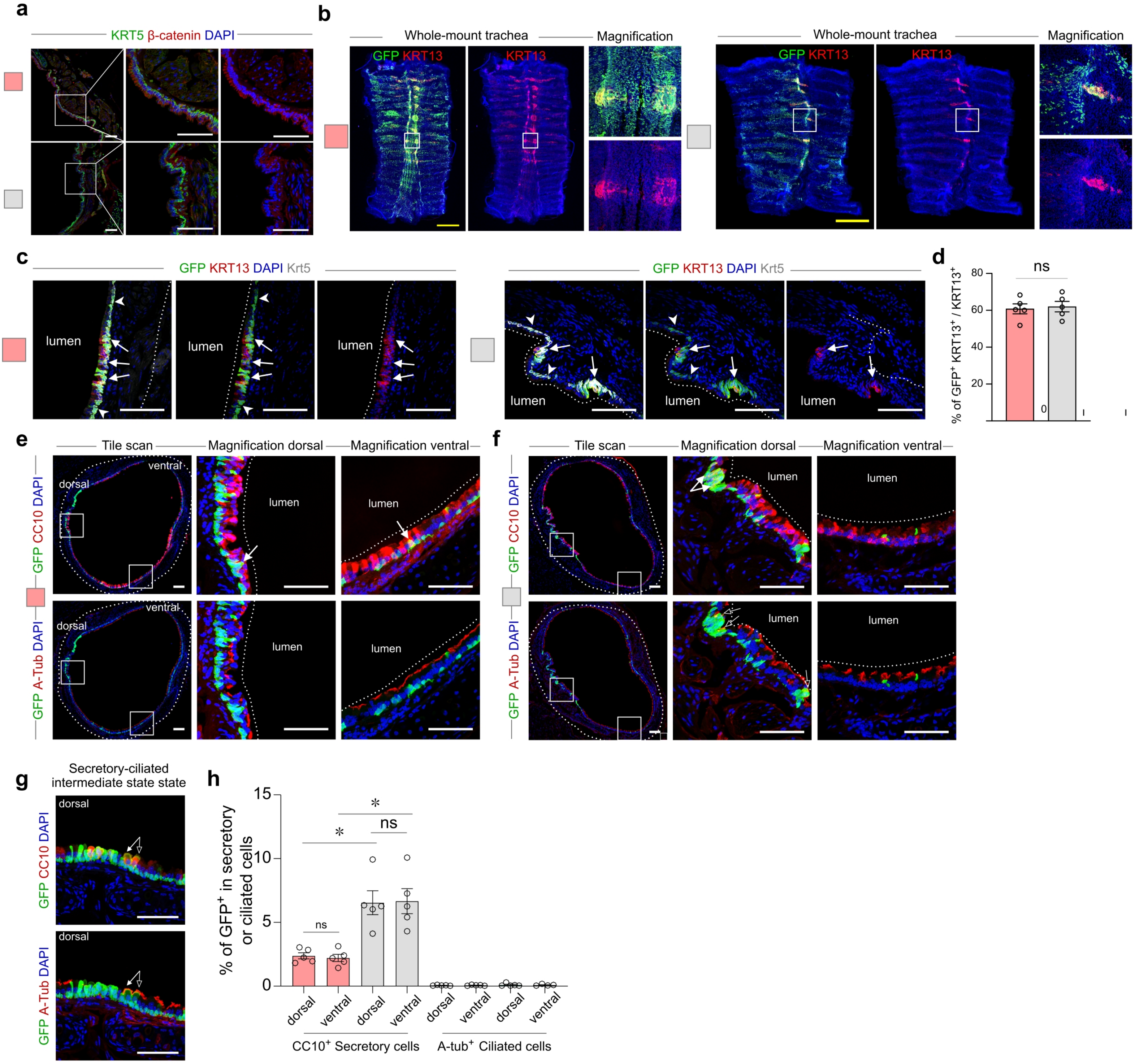

a
KRT5 β-catenin DAPI
b
Whole-mount trachea
Magnification
GFP KRT13
KRT13
Whole-mount trachea
Magnification
GFP KRT13
KRT13
c
GFP KRT13 DAPI Krt5
lumen
lumen
lumen
GFP KRT13 DAPI Krt5
lumen
lumen
lumen
d
ns
% of GFP+ KRT13+ / KRT13+
80
60
40
20
0
e
Tile scan
Magnification dorsal
Magnification ventral
GFP CC10 DAPI
GFP A-Tub DAPI
dorsal
ventral
lumen
lumen
f
Tile scan
Magnification dorsal
Magnification ventral
GFP CC10 DAPI
GFP A-Tub DAPI
lumen
lumen
g
Secretory-ciliated intermediate state state
dorsal
GFP CC10 DAPI
GFP A-Tub DAPI
h
% of GFP+ in secretory or ciliated cells
15
10
5
0
*
ns
*
ns
dorsal
ventral
CC10+ Secretory cells
A-tub+ Ciliated cells

Supplementary Figure 7: Wnt/β-catenin signaling regulates basal cell proliferation dynamics.

a Immunostaining for β-catenin in basal cells ($KRT5^+$) of *β-catenin*$^{fl/fl}$ vs. control (*β-catenin*$^{fl/+}$) mice 4 weeks post-Tam. **b** Whole-mount immunostaining for GFP and KRT13 on BC-ProTracer of *β-catenin*$^{fl/fl}$ vs. control (*β-catenin*$^{fl/+}$) mice 4 weeks post-Tam. **c** Co-staining for GFP, KRT13, and KRT5 on trachea sections of control and cKO mice. Arrows indicate $KRT13^+GFP^+$ cells, arrow heads indicate $KRT13^-GFP^+$ basal cells. **d** Quantification of the proportion of GFP^+KRT13^+ cells in hillock cells. **e, f** Co-staining for GFP, CC10 (**f**, secretory), and A-Tub (**g**, ciliated). **g** Rare GFP^+CC10^+A-Tub^+ intermediate cell state observed in the control group. **h** Quantification of GFP^+ secretory and ciliated cells.

Statistical analysis: Unpaired t-test; ✻ $P \leqq 0.05$; ns, $P > 0.05$. Data represent mean ± SEM. Scale bars: yellow, 1mm; white, 100 µm.
